# Selective Detection and Automated Counting of Fluorescently-Labeled Chrysotile Asbestos Using a Dual-Mode High-Throughput Microscopy (DM-HTM) Method

**DOI:** 10.3390/s130505686

**Published:** 2013-05-02

**Authors:** Myoung-Ock Cho, Hyo Mi Chang, Donghee Lee, Yeon Gyu Yu, Hwataik Han, Jung Kyung Kim

**Affiliations:** 1 Department of Mechanical Engineering, Graduate School, Kookmin University, Jeongneung-ro 77, Seongbuk-gu, Seoul 136-702, Korea; E-Mails: fallslover@naver.com (M.-O.C.); hiro0308@hanmail.net (D.L.); 2 Department of Chemistry, Graduate School, Kookmin University, Jeongneung-ro 77, Seongbuk-gu, Seoul 136-702, Korea; E-Mails: hyomi_2001@nate.com (H.M.C.); ygyu@kookmin.ac.kr (Y.G.Y.); 3 School of Mechanical Systems Engineering, Kookmin University, Jeongneung-ro 77, Seongbuk-u, Seoul 136-702, Korea; E-Mail: hhan@kookmin.ac.kr; 4 Department of Integrative Biomedical Science and Engineering, Graduate School, Kookmin University, Jeongneung-ro 77, Seongbuk-gu, Seoul 136-702, Korea

**Keywords:** asbestos, chrysotile, DksA, high-throughput microscopy, dual-mode imaging, reflection, fluorescence, image processing and analysis, automated counting

## Abstract

Phase contrast microscopy (PCM) is a widely used analytical method for airborne asbestos, but it is unable to distinguish asbestos from non-asbestos fibers and requires time-consuming and laborious manual counting of fibers. Previously, we developed a high-throughput microscopy (HTM) method that could greatly reduce human intervention and analysis time through automated image acquisition and counting of fibers. In this study, we designed a dual-mode HTM (DM-HTM) device for the combined reflection and fluorescence imaging of asbestos, and automated a series of built-in image processing commands of ImageJ software to test its capabilities. We used DksA, a chrysotile-adhesive protein, for selective detection of chrysotile fibers in the mixed dust-free suspension of crysotile and amosite prepared in the laboratory. We demonstrate that fluorescently-stained chrysotile and total fibers can be identified and enumerated automatically in a high-throughput manner by the DM-HTM system. Combined with more advanced software that can correctly identify overlapping and branching fibers and distinguish between fibers and elongated dust particles, the DM-HTM method should enable fully automated counting of airborne asbestos.

## 1. Introduction

Asbestos is a type of silica compound comprised of microscopic bundles of silicate fibers that can easily become airborne [1]. Asbestos has several advantages, including durability, heat resistance, and flame resistance; therefore, it has been used widely as a construction material. However, since it is reported that accumulation of asbestos in the body causes serious diseases such as lung cancer, malignant mesothelioma, and other respiratory symptoms, its use is prohibited in most developed countries. Despite regulations, asbestos contamination still remains to be a common problem and many people die from exposure to asbestos worldwide [2]. There are several established methods to detect asbestos, including phase-contrast microscopy (PCM) [3], polarized microscopy (PLM) [4], X-ray diffraction analysis and transmission electron microscopy (TEM) [5]. Although PCM is simple and cost-effective compared to other methods, it has some limitations. PCM cannot detect thin fibers with diameters less than 0.25 μm due to a resolution limit, and it cannot clearly identify certain types of asbestos [6]. However, asbestos fiber dimension is an important factor in determining respiratory disease risk. Both lung cancer and asbestosis have been strongly associated with exposure to thin asbestos fibers (<0.25 μm) [7]. In addition, the PCM method gives inaccurate results due to the subjective analysis of the operator. Relatively accurate methods, including EM, also require trained experts and expensive equipments.

Thus, innovative techniques for detecting asbestos are being studied in many areas; for example, automated image analysis techniques to replace the conventional manual counting method have shown remarkable results. These methods are mostly based on the color dispersion of asbestos. Kawabata *et al.* [8] developed a qualitative asbestos detection method by modifying conventional methods, and they attempted to detect only asbestos fibers among many types of particles by using both color dispersion and shape information. The use of refraction phenomena or polarization also allows asbestos to be distinguished from other particles. Moriguchi *et al.* [9] and Nomoto *et al.* [10] attempted to automate the detection of asbestos by “image matching,” which uses dispersion staining to search for color changes between two images in order to identify the asbestos. However, due to the complicated process, their methods required enormous time for image processing and analysis, and inconspicuous color changes remained difficult to detect.

An automated image acquisition system is another indispensable requirement for the development of automated airborne asbestos counting method. In our previous study [11], we developed a high-throughput microscopy (HTM) method and demonstrated the feasibility of automated image acquisition and counting of asbestos-like fibers by comparing HTM results with conventional PCM. The proposed HTM method reduces the analysis time significantly from ∼90 min to <5 min using a batch process executing a list of built-in commands of public domain freeware ImageJ, despite the fact that the field-of-view is enlarged by ∼40-fold from 0.00785 mm^2^ to 0.3185 mm^2^. Accuracy of the HTM method was reasonably comparable to PCM when tested on Proficiency Analytical Testing (PAT) standard asbestos samples. A common limitation of the PCM and HTM methods, both of which are based on bright-field imaging, is their inability to distinguish asbestos from non-asbestos fibrous materials.

In a recent study, Kuroda *et al.* [12] implanted a biotechnology into asbestos detection technique. They used DksA, a chrysotile-binding protein, to quantitatively detect chrysotile, based on a colorimetric method by using DksA-AP, and they also used DksA-GFP for fluorescence imaging. The same research group used a new protein, GatZ, to distinguish amosite and crocidolite, which are members of the amphibole mineral group, from chrysotile which belongs to the serpentine mineral group [13]. These protein-based methods have distinct advantages of detecting specific types of asbestos over existing methods by tagging fluorescent dyes to those proteins bound to asbestos fibers. Mossman *et al.* [14] proposed that the biological effects from various kinds of asbestos fibers should be considered individually due to their different properties including chemical composition, morphology, and durability. Selective detection methods using specific proteins might solve these problems.

Here, we aim to improve the selectivity of asbestos detection using the chrysotile-binding protein extracted by the recombinant protein production method together with a newly designed dual-mode high-throughput microscopy (DM-HTM) system, which can obtain both fluorescence and reflected light images by fast scanning of an asbestos sample slide followed by automated image processing and analysis based on ImageJ software for enumeration of the asbestos fibers.

## 2. Materials and Methods

A fluorescent dye-tagging method was used for chrysotile visualization. Fluorescence imaging techniques of asbestos samples using fluorescent bio-probes have been basically developed by Kuroda *et al.* [12] and Ishida *et al.* [13]. We used the DksA protein for HTM analysis to enhance the sensitivity and selectivity of detecting chrysotile asbestos, which has been widely used commercially in many countries. We also attempted automatic enumeration of chrysotile fibers tagged with a fluorescently-labeled protein as well as total fibrous materials in a high-throughput manner, by the DM-HTM device. The results of manual counting were compared with those from the automated analysis of asbestos images taken in both reflection and fluorescence modes.

### 2.1. Extraction of Chrysotile-Adhesive Protein

DksA, comprised of 151 amino acids, is an α-helical protein that can be divided into three distinct structural fragments. DksA is expected to bind to RNA polymerase (RNAP) and is required for control of rRNA transcription by ppGpp *in vivo* [15]. Genomic DNA from *Escherichia coli* (*E*. *coli*) was used as a template and amplified by the polymerase chain reaction (PCR) by using the primer set shown in Table 1. Amplified DNA fragments and the pET21-α plasmid were digested with *Nhe*I and *Bam*HI, respectively, and the DNA was inserted into the *Nhe*I and *Bam*HI sites of pET21-α. DNA inoculated with the vector was transformed into DH5-α competent cells and incubated for 16 h at 36 °C on a Luria-Bertani (LB) agar plate.

**Figure 1.**
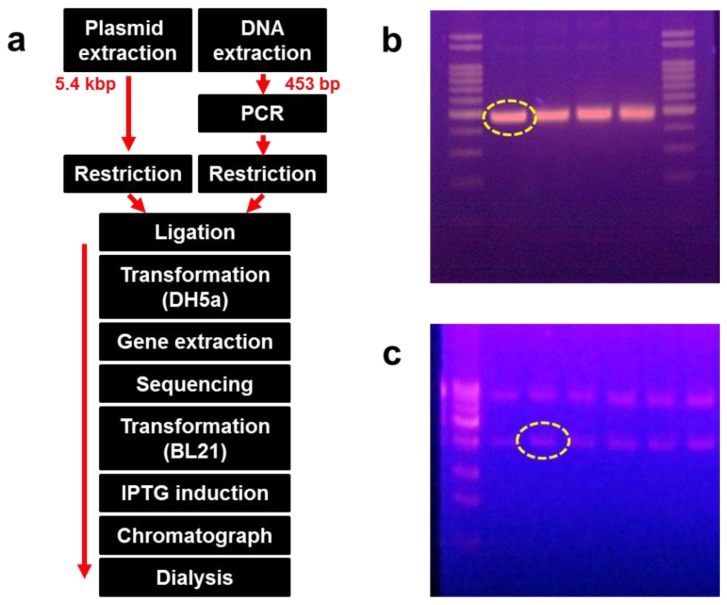
The procedure for extraction of chrysotile-adhesive protein DksA. (**a**) A flow chart for recombinant protein production. (**b**) Amplified DNA fragment followed by polymerase chain reaction using *E. coli* genomic DNA (dotted circle: size = 453 bp). (**c**) pET21-α plasmid vector loaded on an agarose gel (dotted circle: size = 3∼3.5 kbp).

Recombinant DNA was extracted from the bacterial colony, and its sequence was identified. The recombinant DNA was transformed again into BL21 (DE3) cells and incubated for 16 h on an LB agar plate at 36 °C. Some colonies were extracted and cultured in LB medium, followed by induction with 0.1 mM IPTG after growing up to 0.4 at OD_600_. The transformed *E. coli* was lysed using a microfluidizer (M-110P; Microfluidics, Newton, MA, USA). The supernatant was collected following centrifugation at 10,000 × *g* at 4 °C for 20 min. We confirmed that the protein was expressed in the soluble form after separation on a 12% SDS-PAGE gel. The supernatant was also passed through a Ni-NTA His-tag affinity chromatography column (Clontech, Mountain View, CA, USA) and dialyzed using a commercial dialysis kit (20-kD Slide-A-Lyzer Dialysis Cassette; Thermo Scientific, Rockford, IL, USA). Figure 1(a) shows the overall process of recombinant protein production, whereas Figure 1(b,c) show the size and amount of the amplified DNA fragments and plasmid, respectively.

### 2.2. Dual-Mode HTM Setup and Image Acquisition

**Figure 2.**
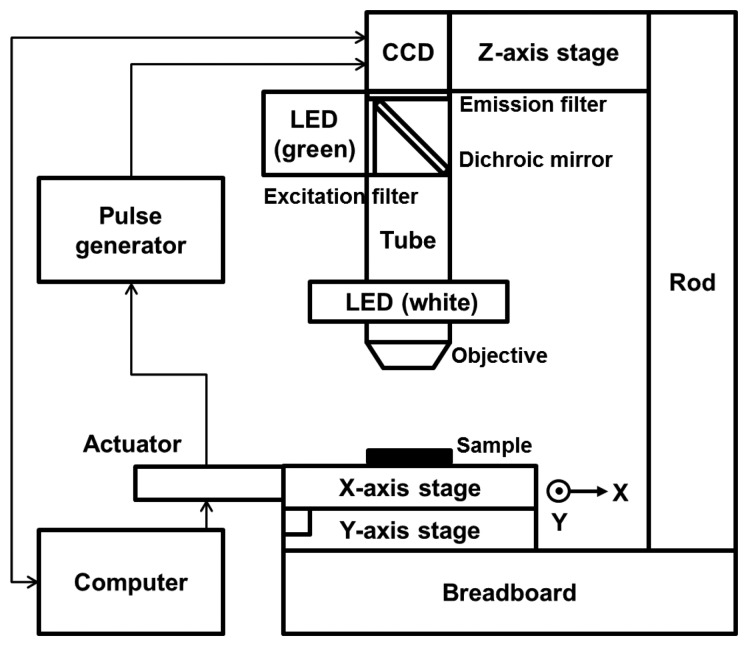
A schematic of DM-HTM system for both reflection and fluorescence imaging.

We modified the configuration of the HTM device partially so that the device could detect a fluorescence signal. Two linear stages (M-426A; Newport, Irvine, CA, USA) were cross-connected on an optical breadboard, and 2 linear actuators were connected to the stages to allow motion in the x- and y-directions. An objective lens (NT36-132; Edmund Optics, Barrington, NJ, USA) and a charge-coupled device (CCD) camera (IMB-20FT; imi tech, Anyang, Korea) were equipped on both sides of a 160 mm scope tube. A brightness-controllable, circular light-emitting diode (LED) was set around the objective. The epi-illumination configuration thus enabled HTM to acquire reflected light images of opaque asbestos samples with good contrast by reducing the background intensity. Several optical filters were added to the basic composition of HTM to allow fluorescence imaging. An emission filter (ET605/70m; Chroma, Bellows Falls, VT, USA) and a dichroic mirror (T565lpxr; Chroma) were used between the CCD camera and the scope tube, and an excitation filter (ET545/25x; Chroma) was added perpendicularly to the circular LED. A green LED (TouchBright X-3; Live Cell Instrument, Seoul, Korea) was used as a fluorescence light source so that the light filtered through the excitation filter excited the fluorescent dye on the asbestos and the emitted fluorescence could be detected. Figure 2 shows a schematic of our modified HTM device for dual-mode reflection and fluorescence imaging.

The stages were automatically controlled by an application software, Zaber Console (Zaber Technologies, Vancouver, Canada). We used a trigger system to acquire asbestos sample images automatically according to the motion of the stages, which traveled a preset distance in each direction every second. We used a custom-made signal conductor control box (Board Lab, Incheon, Korea) for this work. The signals from the stage actuators were sent to the CCD camera with 0.5 s delay time after amplified in the control box. The travel distance was set to 650 μm in the x-direction and 490 μm in the y-direction in accordance with the area of the display window in an image acquisition software (CamViewer; imi tech). After obtaining 108 reflection images within a slide sample in the area of 5 × 5 mm^2^, fluorescence images were taken at the same locations with the green LED on.

### 2.3. Verification of Chrysotile-Adhesive Protein

Three types of asbestos (chrysotile, amosite and crocidolite) were used to verify the adhesive property of the DksA protein. Two hundred microliters of dialyzed protein solution was mixed with 0.6 g of each asbestos sample under high salt conditions (100 mM NaCl), and the mixtures were incubated at 4 °C for 30 min. Following centrifugation at 12,000 rpm for 1 min, the pellets were washed twice with phosphate-buffered saline. After boiling for 10 min, the lysates were separated on a 12% SDS-PAGE gel.

### 2.4. Detection of Fluorescently-Labeled Chrysotile Fibers

We validated the performance of the fluorescence imaging mode of our modified HTM system using fluorescent beads (F8827; Invitrogen, Carlsbad, CA, USA) with a nominal diameter of 2 μm. We obtained the images of the fluorescent beads injected into the plastic sample chamber of known volume and counted them automatically using the image analysis software. The theoretical number of beads per milliliter can be estimated by the following mathematical formula:

(1) $$\begin{array}{c} \text{Number of microspheres}/\text{mL}=6C\times 10^{12}/\rho \pi \emptyset^{3}\\ ∁:\text{concentration of suspended beads}\;[\text{g}/mL]\\ \rho :\text{density of polymer}\;[\text{g}/mL]\\ \emptyset :\text{diameter of microspheres}\;[\mu \text{m}] \end{array}$$

The purified protein was tagged with a fluorescent dye using a commercialized kit (Alexa Fluor 555 Protein Labeling Kit; Invitrogen) to visibly confirm the protein-bound asbestos. The concentration of dye-tagged protein was adjusted to 100 μg/mL. Three types of asbestos samples (0.2 g each) were mixed with 5 μL of dye-tagged protein in three sample tubes, while a second group of tubes was prepared as control asbestos samples mixed with pure dye without the protein. The sample tubes were incubated for 30 min after adding 100 mM NaCl to each tube to prevent nonspecific reactivity. Then the samples were washed three times with a washing buffer (0.1 M sodium carbonate, 1% Tween 80, and 1% polyethyleneimine), dropped on a slide glass, sealed with colorless nail-polish to avoid drying, and observed under a fluorescence microscope (BX51; Olympus, Tokyo, Japan). For quantitative analysis, the chrysotile sample suspension was serially diluted in four steps and three slides were prepared at each sample concentration. One hundred and eight images acquired at the individual slide in the reflection or fluorescence mode were analyzed to produce the total number of fibers, and an averaged value of three automatic counts was reported as fiber count per unit area. We also used mixed samples of chrysotile and amosite to assess the enhanced selectivity of our proposed method. The fluorescently-labeled chrysotile sample suspension with a concentration of 200 μg/mL was serially diluted in four steps with an amosite suspension. We obtained both reflection and fluorescence images of the mixed asbestos samples using the DM-HTM device and enumerated fibers in each image automatically as described in the following section.

### 2.5. Image Processing and Analysis

We used java-based public domain free software ImageJ (National Institutes of Health, Bethesda, MD, USA) as an image processing and analysis program and applied additional plugins to detect asbestos effectively. Several steps for image processing and analysis were applied to a stack of sample images in our previous study [11], such as “Subtract Background,” “Auto Local Threshold,” “Smooth,” and “Analyze Particles.” The irregularity of the background brightness in the image was reduced through the “Subtract Background” process, and we used “Auto Local Threshold” to correct background illumination by varying a window of radius “r” around the image. These images were set to 0 ∼ 0.663 in “Circularity” for the “Analyze Particles” process to detect fibers longer than 5 μm with an aspect ratio ≥3 in accordance with the counting rules stated in NIOSH 7400 [3]. The circularity defined as 4π(area/perimeter^2^) is a function of the aspect ratio (*α*), the ratio between the long axis and the short axis of the ellipse, as given below:

(2) $$\text{Circularity}=4\pi (\text{area}/{\text{perimeter}}^{2})=4\alpha /{\left\{3(1+\alpha )-\sqrt{\left(3+\alpha )(1+3\alpha \right)}\right\}}^{2}$$

In our previous study [11], the upper limit of the circularity was incorrectly set to 0.33 which corresponds to *α* ≈ 7. Although we cannot exclude the possibility of omitting fibers with 3 ≤ *α* < 7 in the automatic counting process, it would have not affected the result significantly because most asbestos samples used in our prior study had long and thin fibrous shapes with *α* ≥ 7. Here we tried to enumerate all the fibers with *α* ≥ 3 in line with the NIOSH 7400 method while excluding elliptical non-asbestos particles by setting a strict range of input parameter “Size” for the “Analyze Particles” process. In order to eliminate small-sized particles and large debris, “Size,” represented as pixel squares, was set according to the minimum and maximum area of asbestos in the images. We analyzed hundreds of both reflection and fluorescence images by running an in-house written macro which carried out a series of built-in commands consecutively to apply a pre-determined optimal set of parameters. The total fiber count can be converted into fiber density (f/mm^2^) or fiber concentration (f/cc) for displaying results.

### 2.6. Manual Counts by A Human Researcher

Automatic counting results from the DM-HTM method were compared with manual counts determined by a human researcher. Three slides were prepared at each concentration of serially diluted asbestos samples and images were taken at two areas per slide. A human researcher counted the total number of fibers in each set of 50 images out of 108 reflection images obtained in one area, and an average value of two manual counts was used for computing the fiber density.

## 3. Results and Discussion

### 3.1. Protein Expression

**Figure 3.**
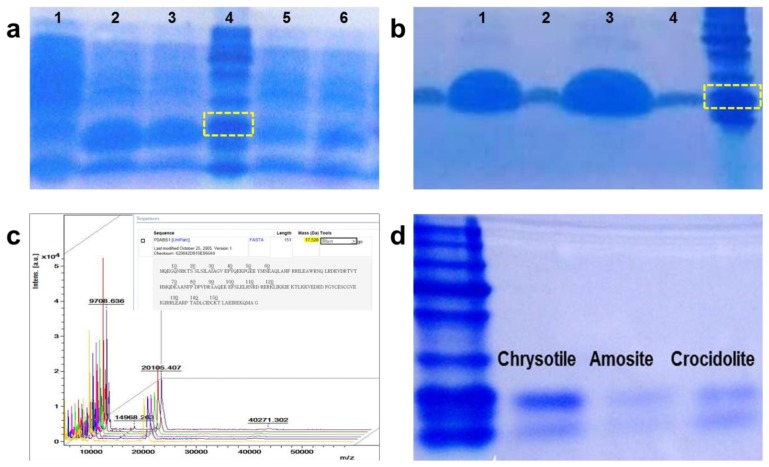
Purification of recombinant protein. (**a**) Result of IPTG induction test. Lane 1, before induction; Lane 2, 0.1 mM IPTG at 37 °C for 3 h; Lane 3, 0.5 mM IPTG at 37 °C for 3 h; Lane 4, protein marker (size = 24 kD); Lane 5, 0.1 mM IPTG at 18 °C for 13 h; and Lane 6, 0.5 mM IPTG at 18 °C for 13 h. (**b**) Results of protein purification. Lane 1 and Lane 3 are the first fractions of eluted protein through the purification column, and Lane 2 and Lane 4 are the third fractions. (**c**) Result of peptide mapping of the recombinant protein. (**d**) Verification of the properties of DksA adhered to chrysotile.

We combined the chrysotile-adhesive protein, DksA, with the DM-HTM platform to enhance the efficiency of asbestos detection, and demonstrated that selective detection of chrysotile is feasible. Expression and purification of DksA protein were conducted according to the method reported by Kuroda *et al.* [12], with the following modifications. The optimized condition for protein expression was obtained as proved in Figure 3(a), which indicates that the sample in the Lane 2 incubated for 3 h at 37 °C after 0.1 mM IPTG induction shows a greater protein yield. The protein was purified using Ni-NTA His-tag affinity chromatography and separated on a 12% SDS-PAGE gel.

Figure 3(b) shows an amount of DksA and its size having a purity more than 90%. After dialysis, the final purified protein was sent for N-terminal sequencing and the exact molecular weight was determined by MALDI-TOF, as shown in Figure 3(c). The size and the concentration of the protein were 20 kD and 2 μg/μL, respectively. We tested the protein against three types of asbestos—chrysotile, amosite and crocidolite—to check whether the purified protein bound only to chrysotile. The asbestos samples were incubated with the protein solution for 30 min and separated by boiling. After centrifugation, the supernatant was loaded onto a 12% SDS PAGE gel. Figure 3(d) shows the presence of DksA attached to the asbestos samples. Although the protein was slightly eluted from both amosite and crocidolite, it mostly bound to chrysotile, implying that the protein strongly and specifically attaches to the chrysotile fibers.

### 3.2. Selective Detection of Chrysotile by Fluorescence Imaging

**Figure 4.**
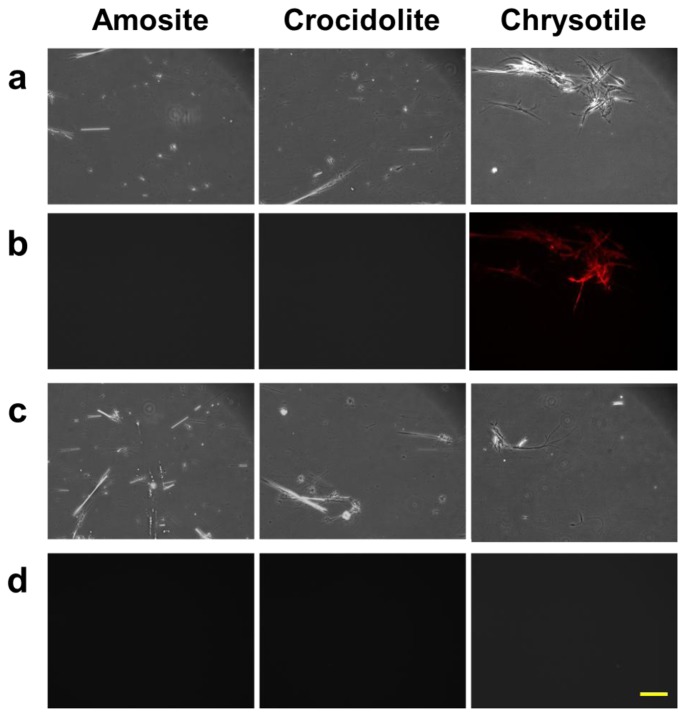
Protein binding test performed for three types of asbestos samples. (**a**) Phase contrast and (**b**) fluorescence images (pseudo-colored red) of asbestos fibers tagged with fluorescently-labeled DksA protein. (**c**) Phase contrast and (**d**) fluorescence images of asbestos fibers treated with fluorescent dye only without DksA protein. Bar = 100 μm.

We also studied the properties of the protein bound to chrysotile through a conventional fluorescence microscopy. The three types of asbestos, chrysotile, amosite and crocidolite, were incubated with the fluorescent dye-labeled DksA protein, and observed under the fluorescence microscope. A second control group of the asbestos samples incubated with the pure dye was also observed to check the nonspecific reactivity. Figure 4 shows the result of the fluorescently-labeled protein and asbestos binding assay. Amosite and crocidolite can be seen in the phase contrast mode (Figure 4(a,c)), but invisible in the fluorescence mode (Figure 4(b)). Chrysotile can be observed more clearly in the fluorescence mode without background noise as shown in Figure 4(b).

Fluorescence signal was not detected in any type of asbestos samples incubated with pure dye solution (Figure 4(d)), indicating that there was no nonspecific reactivity by the fluorescent dye and that the DksA protein bound specifically to the chrysotile fibers. The phase-contrast and fluorescence images acquired with the conventional fluorescence microscopy can be used as reference images for subsequent comparison with reflection and fluorescence images obtained by the DM-HTM system. The specificity of DksA binding to chrysotile has also been tested against all five remaining types of asbestos as well as ten different kinds of non-asbestos fibers by Ishida *et al.* [13,16].

**Figure 5.**
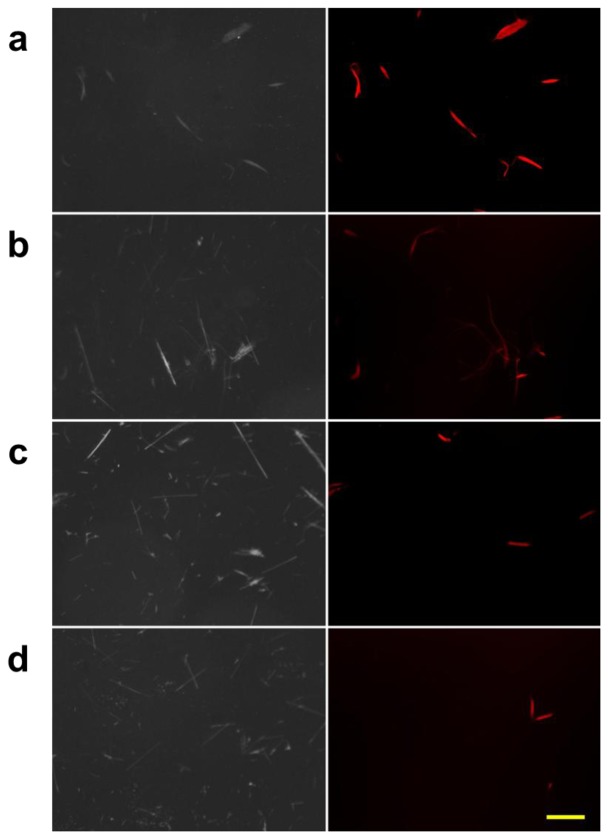
Reflection and fluorescence images of mixed samples of chrysotile and amosite at various concentration ratios. Reflection (left column) and fluorescence images (right column; pseudo-colored red). Concentration ratio of chrysotile to amosite: (**a**) 200/0, (**b**) 100/100, (**c**) 50/150, and (**d**) 25/175 μg/mL. Bar = 100 μm.

A further test was conducted to confirm the selectivity of the protein against chrysotile. We created mixed samples of chrysotile and amosite at different concentration ratios, and then observed them using the DM-HTM device. We acquired both reflected light images and fluorescence images of the mixed asbestos sample at the same positions on the slide. The images of the mixed asbestos samples are displayed in Figure 5.

As the amount of chrysotile was decreased with the increasing amount of amosite, it was difficult to detect chrysotile in the reflection mode at low concentrations of chrysotile owing to the interference by amosite. However, it was straightforward to identify the chrysotile fibers in the fluorescence images.

### 3.3. Automated and High-Throughput Counting of Chrysotile Using DM-HTM

**Figure 6.**
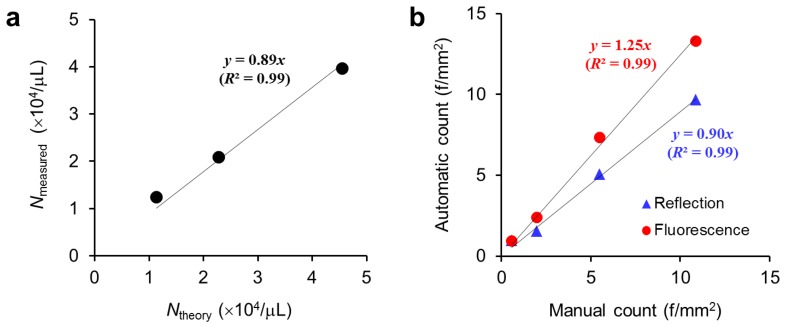
Validation of serially diluted microbead and fiber counts by automated image processing and analysis. (**a**) Comparison of theoretical (*N*_theory_) and measured (*N*_measured_) numbers of microspheres in fluorescence mode of DM-HTM method. (**b**) Comparison of chrysotile asbestos fiber counts determined by DM-HTM and manual counting.

Our DM-HTM device was able to acquire both reflection and fluorescence images consecutively at the same positions of the sample slide. Additional light source and fluorescence filters were added to the HTM device, and the scan pathway was changed to the regression mode in order to return to the position where the first reflection image was taken, and to start acquiring fluorescence images. The capabilities of the DM-HTM system were tested by running the macro that automates a series of built-in commands and plugins provided by the ImageJ. Figure 6(a) shows the result of counting the serially diluted fluorescent microbeads. The slope of the linear regression line through the origin is 0.89 (*R*^2^ = 0.99) denoting that the measured number of particles (*N*_measured_) is about 10% less than the theoretical number of particles (*N*_theory_), presumably due to sample loss during pipetting and uneven distribution of particles in the chamber. Chrysotile samples that were diluted in four steps were prepared to prove the possibility of enhancing the sensitivity of detecting asbestos fibers in the fluorescence mode. We acquired reflection images of highly diluted samples at low intensity level of illumination deliberately making the images have lower contrast. The results from the analysis of both reflection and fluorescence images by DM-HTM were compared with the results from the manual counting method described in Section 2.6 and presented in Figure 6(b). Manual count represents the total number of fibers enumerated from a set of 50 reflection images by a human researcher and is considered to be the gold standard in this study.

According to the slopes of the linear regression lines in Figure 6(b), we found that the automatic counts from reflection and fluorescence image analyses were 10% less and 25% greater than the manual counts, respectively. Although neither method accurately reproduces the manual counting, the correlation with the manual counts is higher for the reflection image analysis. This difference could be caused by uncounted small fibers and fibers with low contrast against the background during manual and automatic analyses of the bright-field reflection images (not shown here). Those undetectable small and low contrast chrysotile fibers can be seen and identified more sensitively in the fluorescence images resulting in automatic counts biased by 25% from the manual counts. The extent of bias is not peculiar to the mode of imaging but dependent on the quality of sample images and image processing scheme as well. Therefore standardization of the testing procedures from sample preparation to image acquisition and analysis is necessary to reduce the bias or to render the bias reproducible. The contrast of detecting asbestos fibers in the bright-field mode can be greatly enhanced with a differential interference contrast microscopy as reported in a recent study [17].

**Figure 7.**
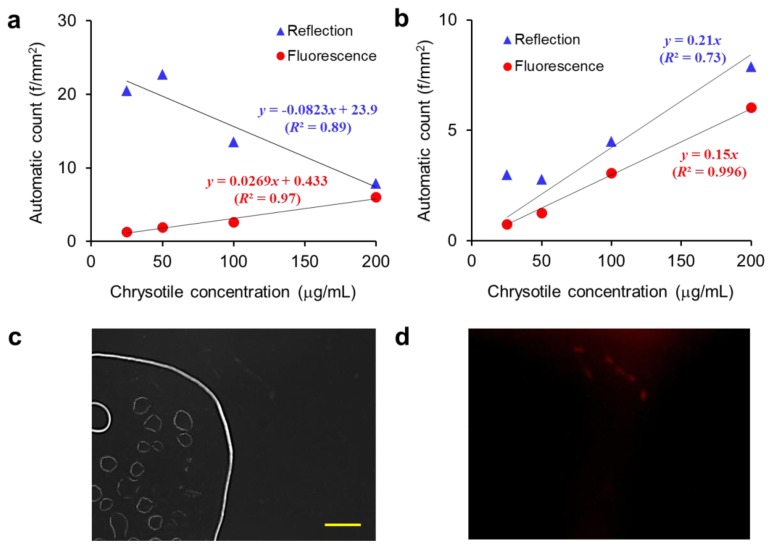
Results from analyses of reflection and fluorescence images. (**a**) Automatic fiber counts of the mixed asbestos samples (chrysotile and amosite) against chrysotile concentrations. (**b**) Automatic fiber counts of chrysotile samples against chrysotile concentrations. Representative reflection image (**c**) with high background noise level and corresponding fluorescence image (pseudo-colored red) (**d**). Bar = 100 μm.

We also evaluated our DM-HTM with mixed asbestos samples as described in Section 2.4. Representative reflection and fluorescence images of the mixed chrysotile and amosite are given in Figure 5. The contrast of the reflection images taken at normal illumination level is much higher than those used for Figure 6(b) and the background noise such as debris and air bubble is noticeable in some images (Figure 7(c)).

Figure 7(a) shows the automatic counts of the mixed asbestos samples against chrysotile concentrations. Since the amosite concentration is inversely related with the chrysotile concentration and the size of amosite is much smaller than chrysotile in the mixed sample, the amount of amosite is greater than chrysotile. Therefore the total fiber count from the reflection image analysis is decreased with increasing chrysotile concentration. However, since only the chrysotile fibers are visible in the fluorescence mode, the number of fibers determined by fluorescence image analysis is directly proportional to the chrysotile concentration.

Figure 7(b) shows the automatic counts of chrysotile samples against chrysotile concentrations. Reflection image analysis resulted in fluctuation and overestimation of the fiber counts in the sample slide where air bubbles were formed, by erroneously recognizing their menisci as fibers. In contrast, the fiber count from fluorescence image analysis is linearly increased with the chrysotile concentration regardless of background noise level in the sample slide. Non-fluorescent debris and bubble images appeared in the reflection image are removed completely in the fluorescence image as exhibited in Figure 7(c,d). This tendency to “over count” has not been considered in the existing PCM method that is based on manual counting in bright-field mode by human naked eye. We may reveal and compensate those biased errors accompanied by manual counting through our DM-HTM method.

We enhanced the sensitivity and selectivity of the HTM method to detect chrysotile exclusively from low-concentration samples containing small and thin fibers and asbestos of different types by the fluorescently-labeled chrysotile-adhesive protein and the DM-HTM system. In particular the background noise, which is often considered to be fibers in bright-field imaging mode, was reduced by approximately 90% in the fluorescence imaging mode. It was reported that chrysotile fibers that are as thin as 30–35 nm can be detected using Cy3-labeled DksA protein [16]. It is promising that the proposed DM-HTM method should realize fully automated counting of airborne asbestos if combined with more advanced software that can identify overlapping and branching fibers and distinguish between fibers and oval-shaped debris.

## 4. Conclusions

We have improved the selectivity of asbestos detection by using the chrysotile-binding protein extracted by the recombinant protein production method together with our newly designed dual-mode high-throughput microscopy (DM-HTM) system. We demonstrated that our DM-HTM method can accomplish high-throughput identification and enumeration of fluorescently-labeled chrysotile fibers exclusively in the fluorescence imaging mode as well as total fibers in the reflection mode with fast scanning of asbestos sample slides followed by automated image processing and analysis. We also proved that the sensitivity is enhanced for the samples with tiny asbestos fibers that are not detected in low contrast bright-field images. The DM-HTM method can be improved substantially to enable the fully automated counting of airborne asbestos by combining with more advanced image processing technique that can correctly identify overlapping, cross-linking and branching fibers and properly differentiate fibers from elongated dust particles. Integration of DM-HTM with a new sampling and pretreatment procedure will extend the application of the protein-based method to on-site and real-time detection of airborne asbestos.

## Figures and Tables

**Table 1.** A set of sequencing primers.

**Primer**	**DNA Sequence**
DksA-forward	GGA ATT CGC TAG CAT GCA AGA AGG AAA CCG
DksA-reverse	GAG CCG TTG GAT CCC CGC CAG CCA TCT GTT TTT CGC
